# Is functional training an efficient approach to improve body composition in older people? A systematic review

**DOI:** 10.3389/fphys.2023.1156088

**Published:** 2023-06-19

**Authors:** Marcos Raphael Pereira Monteiro, Alan Pantoja Cardoso, Antônio Gomes de Resende-Neto, Alan Bruno Silva Vasconcelos, Enilton Aparecido Camargo, Luis Alberto Gobbo, José Luis Maté-Muñoz, Juan Ramón Heredia-Elvar, David George Behm, Marzo Edir Da Silva-Grigoletto

**Affiliations:** ^1^ Department of Physiology, Federal University of Sergipe, São Cristóvão, Brazil; ^2^ Department of Physiotherapy, Federal University of Sergipe, Lagarto, Brazil; ^3^ Department of Physical Education, Federal University of Sergipe, São Cristóvão, Brazil; ^4^ Department of Medicine, Federal University of Sergipe, São Cristóvão, Brazil; ^5^ Department of Physical Education, São Paulo State University, Presidente Prudente, Brazil; ^6^ Department of Radiology, Rehabilitation and Physiotherapy, Complutense University of Madrid, Madrid, Spain; ^7^ Department of Physical Activity and Sports Science, Alfonso X El Sabio University, Madrid, Spain; ^8^ School of Human Kinetics and Recreation, Memorial University of Newfoundland, St. John’s, NL, Canada

**Keywords:** aging, exercise, health, body fat distribution, resistance training

## Abstract

**Introduction:** Increases in fat mass and reductions in lean mass are associated with the frailty and mortality of older people. In this context, Functional Training (FT) is an option to increase lean mass and reduce fat mass in older people. Thus, this systematic review aims to investigate the effects of FT on body fat and lean mass in older people.

**Methods:** We included randomized controlled clinical trials, with at least one intervention group that employed FT, with the age of participants ≥60 years; and participants physically independent and healthy. We performed the systematic investigation in Pubmed MEDLINE, Scopus, Web of Science, Cochrane Library, and Google Scholar. We extracted the information and used the PEDro Scale to assess the methodological quality of each study.

**Results:** Our research found 3,056 references with five appropriate studies. Of the five studies, three presented reductions in fat mass, all of them with interventions between three and 6 months, different training dose parameters, and 100% of the sample was composed of women. On the other hand, two studies with interventions between 10 and 12 weeks presented conflicting results.

**Conclusion:** Despite the limited literature about lean mass, it appears that long-term FT interventions may reduce fat mass in older women.

**Clinical Trial Registration:** https://www.crd.york.ac.uk/prospero/display_record.php?RecordID=399257, identifier CRD42023399257

## 1 Introduction

As age advances, changes occur in several systems, altering the body composition and impacting the health of older people (Jafarinasabian et al., 2017; Zullo et al., 2020). In this context, aging can induce a decrease in lean body mass, reducing the number and size of skeletal muscle fibers with a higher prevalence of loss in type II fibers (Essén-Gustavsson and Borges, 1986). This muscle loss averages 0.47% and 0.37% per year for men and women, respectively (Mitchell et al., 2012). Besides this loss in lean mass, the aging process can promote increases in body fat, with consequent fat redistribution to visceral organs and muscle infiltration (Lang et al., 2010; Hunter et al., 2014). These changes in body composition are strongly associated with proinflammatory status, frailty, and mortality (Brown et al., 2017; Reinders et al., 2017; Rossi et al., 2019). In turn, exercise is an attractive choice to delay the effects of aging (Liberman et al., 2017). In this way, the exercise can improve lean mass and reduce fat mass, subsequently impacting in the muscle strength, functionality, proinflammatory status and immune health (Distefano and Goodpaster, 2018; Duggal et al., 2019; Jiang et al., 2019).

Among the training methods, multicomponent training stands out. This type of training is characterized by developing multiple physical capabilities such as strength, endurance, flexibility, and balance, improving functionality, and presenting itself as a highly cost-effective option (Cadore et al., 2013; Bouaziz et al., 2016; Northey et al., 2018; Jorge et al., 2022). Besides working on different physical capabilities, the most recent positions in the literature emphasize the importance of the specificity principle in the training of older people (Behm and Sale, 1993; Forman et al., 2017; Fragala et al., 2019; Izquierdo et al., 2021). On this premise, Functional Training (FT) appears in the literature, presenting exercises with a multicomponent training character, and exploring different movement planes, presenting a multiplanar training character. It is important to note that FT tries to simulate in the training session the demands of the body to perform activities of daily living. In this way, FT considers the principle of specificity by promoting synergistic and integrated adaptations in physical capabilities, stimulating these capabilities in patterns similar to the activities of daily living (La Scala Teixeira et al., 2017; Da Silva-Grigoletto et al., 2020).

By keeping the characteristics of multicomponent training and presenting the need to consider high-specificity exercises, FT is more in line with recent positions in the literature (Da Silva-Grigoletto et al., 2020; Izquierdo et al., 2021). FT appears as a good option for training to promote the health of older people, including increases in strength, muscle power, and functionality (Liu et al., 2014; Da Silva-Grigoletto et al., 2019; Vasconcelos et al., 2020; Aragão-Santos et al., 2021). Although FT promotes improvements in health and functionality in older people, there are still questions about whether FT *per se* can also change body composition, going beyond function and impacting morphology. Based on this query, this systematic review aims to provide a high level of evidence about the effects of FT on body fat and lean mass of older people.

## 2 Materials and methods

### 2.1 Experimental design

This systematic review followed the Preferred Reporting Items for Systematic Reviews and Meta-Analyses (PRISMA) guideline (Page et al., 2020) and was previously registered in the PROSPERO database (registration number: CRD42023399257).

### 2.2 Search strategy

This review included studies that examined the effects of FT on body fat and lean mass of older people. We utilized the Rayyan system to select the studies (Ouzzani et al., 2016). The PICOS strategy adopted was P: Population, older people; I: Intervention, FT; C: Comparison of other exercise or control interventions; O: Outcomes, fat mass or lean mass; S: Study type. Randomized clinical trials.

We performed a computerized systematic literature search in March 2023, including all the previous articles published in the following databases: Pubmed MEDLINE, Scopus, Web of Science, Cochrane Library, and Google Scholar. In particular, in Google Scholar, only 10 initial pages were analyzed. The strategy of research utilized different combinations of the keywords: “Functional Training”; “Functional Exercise”; “Functional Task Exercise”; “Functional Task Training; “Elderly”; “Aged”; “Older People”; “Older Adults” with the Boolean operators “AND” and “OR”. Further, we manually searched the references of the selected articles to add relevant articles to our final database. The strategies performed were described and available as Supplementary Material.

### 2.3 Selection criteria

The following eligibility criteria were adopted based on the PICOS strategy: Population, 1) the age of participants needed to be ≥60 years, and 2) participants needed to be physically independent and healthy; Intervention, 3) have at least one intervention group that employed FT, without nutritional supplementation; Comparison, 4) present other exercise or control intervention group; Outcome, 5) present evaluation of fat mass or lean mass; Study type, 6) be a randomized clinical trial. The exclusion criteria were 1) present a sample that had pathologies affecting functional aspects of movement, such as Parkinson’s disease and stroke, and 2) be short communications, notes, letters, and brief reports. Based on these eligibility criteria, two independent researchers (M.R.P.M. and A.C.P.) performed a screen of the papers analyzing the titles, abstracts, and full texts. Disagreement between the researchers was resolved with a consensus with a third member.

### 2.4 Data extraction

We extracted information about the study population, intervention groups, intervention characteristics (intervention duration, session time, frequency, and intensity), exercises performed in the FT session, evaluation instrument, dependent variables, effect size related to FT, and observed outcome for fat mass and lean mass. All these characteristics were tabulated, and we compared the studies based on this information.

### 2.5 Assessment of methodological quality

The PEDro Scale was used to assess the quality of the studies. The scale involves important points such as properly allocated process, blinding, intention-to-treat analysis, and adequate follow-up. The methodological quality score of the articles is expressed in a score from 0 to 10, with a cut-off score of 6 for high-quality studies (Maher et al., 2003). The cut-off score of 6 was not a criteria point for including studies in this review. Two independent researchers (M.R.P.M. and A.P.C.) performed the quality assessment, and a third researcher was consulted if necessary for disagreement.

## 3 Results

### 3.1 Selection

**FIGURE 1:**
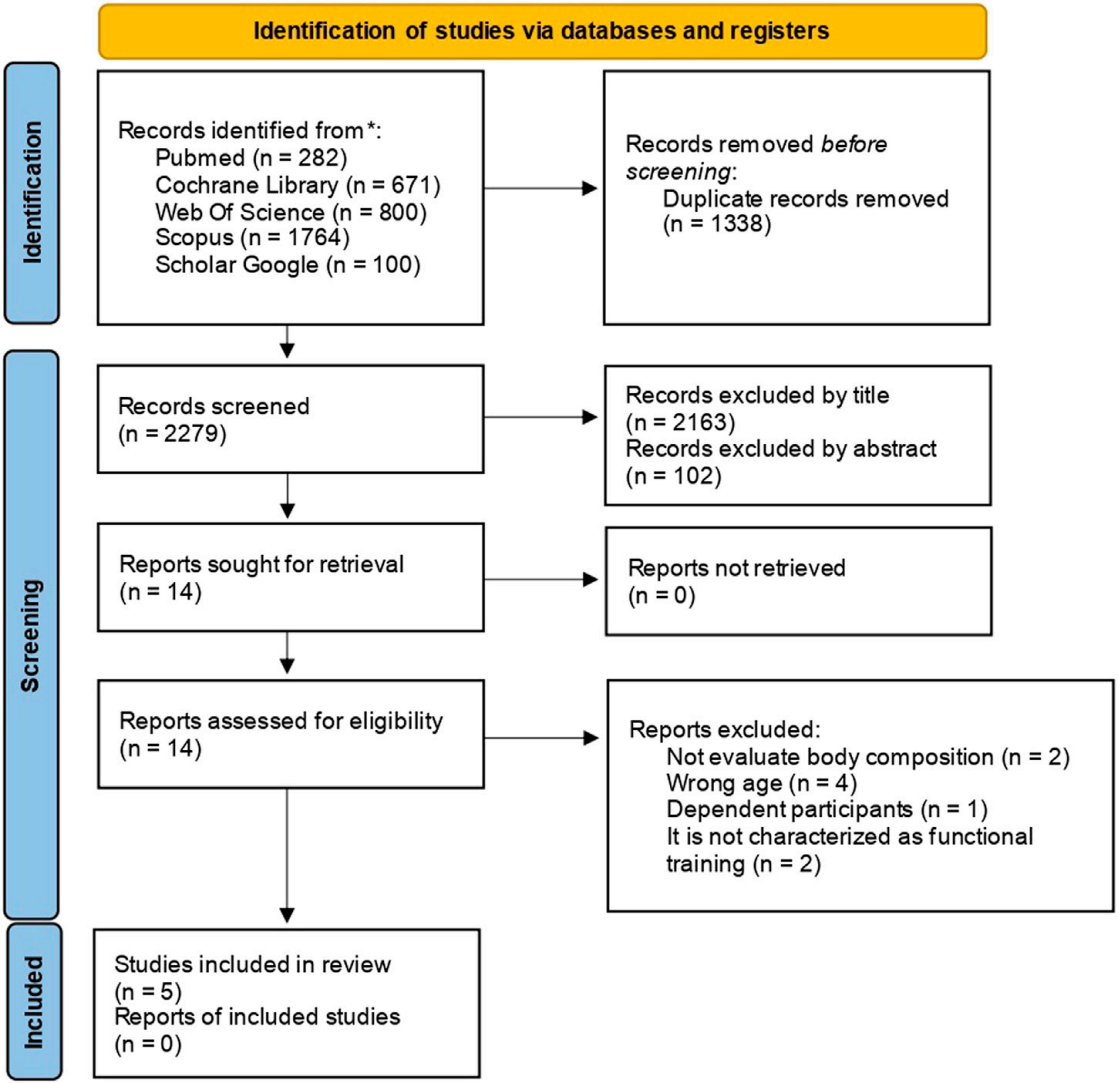
Flowchart represening the systematic review process to include studies.

The initial search found 3,056 references. We removed 1,166 duplicates, and 1890 references were analyzed by title and abstract. Further, we analyzed the 38 articles that remained, and only five complied with the eligibility criteria. Our manual search did not identify other relevant studies to add (Figure 1).

### 3.2 Study characteristics

The main characteristics of the studies were extracted and organized in a table format (Table 1). The studies presented a mean study sample of 44.4 ± 11.2 participants. The study with the smallest sample size was Manini et al. (2007), which presented a sample of 32 participants. The study with the largest sample size was Wiszormiska et al. (2015) which had a sample size of 60 participants. Women made up 98.1% of the sample population. We can see that only one study included older men in the sample, presenting a percentage of 9.4% of the total sample in that study (Manini et al., 2007). The other studies included in this review evaluated only women (Wiszomirska et al., 2015; Resende-Neto et al., 2019a; Resende-Neto et al., 2019b; Mile et al., 2021). Three studies included in our review (Manini et al., 2007; Resende-Neto et al., 2019a; Resende-Neto et al., 2019b) compared FT with protocols of traditional resistance training. Manini et al. (2007) even included a group that alternated between FT and traditional training (TT). Wiszormiscka et al. (2015) compared FT among older with young women. Finally, Mile et al. (2021) compared the effects of FT with and without the administration of ACE inhibitors.

**TABLE 1:** Characteristics of included studies.

Study	n	Women (%)	Intervention groups	Intervention characteristics	Exercises performed	Evaluation instrument	Dependent variables	Effect size for FT in time	Observed outcome
Manini et al. (2007)	32	90.6	FT (n = 10; 78.9 ± 6.7 years; 26.9 ± 5.0 kg/m^2^)	Total time: 10 weeks	FT: Rising from a chair, rising from knelling position, stair climbing, vacuuming a carpet with a weighted cleaner and lifting and carrying a weighted laundry basket.	Dual-energy x-ray absorptiometry - DEXA (Lunar DPX; GE Medical Systems, Waukesha, WI)	Appendicular Fat Mass	Appendicular Fat Mass: −0.02	Only the lean to fat ratio showed significant differences over time, but did not maintain this statistical difference in multiple comparisons
			RT (n = 11; 74.4 ± 10.6 years; 31.6 ± 5.3 kg/m^2^)	Weekly frequency: 2x	RT: Leg Press, leg extension, leg curl, sitting dip, arm curl and shoulder press		Appendicular Lean Mass	Appendicular Lean Mass: 0.15	
			FRT (n = 11; 74.4 ± 7.4 years; 30.9 ± 6.6 kg/m^2^)	Session time: 30–45 min	FRT: FT + RT		Lean to Fat Ratio		
				FT intensity: Progression of task based on complexity					
				RT Intensity: 10RM					
				RFT: FT + RT					
Wiszormiska et al. (2015)	60	100	FT - OW (n = 30; 68.7 ± 7.55 years; 26.6 ± 4.71 kg/m^2^)	Total time: 5 months (≅ 20 weeks)	FT: Lifting one lower limb, performing squat + lifting one lower limb to the side, performing semi-squat + lifting one lower limb to back, lifting lower limb at 90° of knee and hip flexion, stretching to the rear the lower limb, performing adapted plank, performing forward lean position + raising the lower limb	Bioeletrical impedance analysis (BC 420 SMA, Tanita Corporation, Tokyo, Japan)	Percentage of Fat Mass	Percentage of Fat Mass: −0.20	Older women showed statistically increase in lean mass and decrease in fat percentage after training
			FT - YW (n = 30; 20 ± 1.14 years; 21.2 ± 1.72 kg/m^2^)	Weekly frequency: 2x			Percentage of Lean Mass	Percentage of Lean Mass: 0.21	
				Session time: 45 min					
				Intensity: not avaliable					
Resende-Neto et al. (2019a)	47	100	FT (n = 32; 65.28 ± 4.96 years; 29.12 ± 5.67 kg/m^2^)	Total time: 12 weeks	FT: Preparation to movement. Step up/step down movements, alternating waves, medicine ball release on the ground, displacement between cones, linear agility ladder, kettlebell lifting, rowing with suspension tape, sit and lift, horizontal adduction, farmers walk, rowing with elastic, bilateral pelvic elevation, front plank	Bioeletrical impedance analysis (Biodynamic^®^, BIA 310, New York, United States)	Percentage of Fat Mass	Percentage of Fat Mass: −0.29	FT showed a statistically significant reduction in fat percentage, but not in fat mass (kg)
			TT (n = 32; 65.28 ± 4.96 years; 29.13 ± 5.48 kg/m^2^)	Weekly frequency: 3x	TT: Preparation to movement. Continuous walk, smith squatting, horizontal articulated rowing, leg press 45°, vertical supine, leg curl, pulled forward, bilateral standing calf, stiff		Total Fat Mass	Total Fat Mass: −0.10	TT showed a statistically significant increase in lean mass
			SG (n = 15; 64.40 ± 3.68 years; 26.40 ± 4.65 kg/m^2^)	Session time: 45min	SG: Static stretching exercise for the main body parts and relaxation exercises		Total Lean Mass	Total Lean Mass: 0.12	
				Intensity: 8-10RM					
Resende-Neto et al. (2019b)	48	100	FT (n = 32; 65.38 ± 5.11 years; 29.30 ± 5.29 kg/m^2^)	Total time: 12 weeks	FT: Preparation to movement. Medicine ball lauches, displacement with cones, jump on a 10 cm step, coordinated exercises, alternating waves, kettlebell deadlift, rowing with suspension tape, sit to stand, push-ups, farmers walk, elastic band rowing, hip elevation and front plank	Bioeletrical impedance analysis (BC-418MA, Tanita Corporation, Tokyo, Japan)	Percentage of Fat Mass	Percentage of Fat Mass: −0.25	There were no significant differences over time for any of the groups
			TT (n = 32; 65.38 ± 5.11 years; 29.02 ± 5.88 kg/m^2^)	Weekly frequency: 3x	TT: Preparation to movement. Continuous walk, smith squatting, rowing machine, leg press 45°, vertical bench press, leg curl, lat full down, leg press and stiff		Total Lean Mass	Total Lean Mass: 0.10	
			SG (n = 15; 64.19 ± 3.68 years; 26.20 ± 9.29 kg/m^2^)	Session time: 50 min	SG: Static stretching exercise for the main body parts and relaxation exercises				
				Intensity: 8-10RM					
Mile et al. (2021)	35	100	FT (n = 17; 66.55 ± 1.29 years; 26.96 ± 2.63 kg/m^2^)	Total time: 6 months (≅ 24 weeks)	FT: Warm up with treadmill/elliptic trainer/bicycle, TRX squat, TRX single squat, TRX low rows, TRX single arm low rows, TRX push up, TRX push up on one leg, TRX standing hip drop, fitball exercises, stretching	Bioeletrical impedance analysis (InBody 770, InBody, Cerritos, United States)	Total Fat Mass	Total Fat Mass: −0.24	Both groups reduced fat mass and increased lean mass
			FT-ACEI (n = 18; 66.17 ± 1.18 years; 26.33 ± 2.63 kg/m^2^)	Weekly frequency: 3x	FT-ACEI: FT + ACEI administration		Total Lean Mass	Total Lean Mass: <0.01	
				Session time: 50 min					
				Intensity: not avaliable					

FT, Functional training; TT, Traditional training; SG, Stretching group; RT, Resistance training; FRT, Functional + resistance training, OW, Older women; YW, Young women; RM, Repetition maximum; ACEI—ACE, inhibitors.

Training duration averaged 15.6 ± 6 weeks. However, it is important to point out that three studies had a total duration less than or equal to 12 weeks (between 10 and 12 weeks) (Manini et al., 2007; Resende-Neto et al., 2019a; Resende-Neto et al., 2019b), and two other studies had a total duration greater than or equal to 20 weeks (Wiszomirska et al., 2015; Mile et al., 2021). These last two studies with longer duration brought their original information in months of training. The mean training frequency was 2.6 ± 0.54 training sessions per week, always using between 2 and 3 times a week. The session time was between 45 and 50 min, but it is important to point out that it was not presented exactly in the study by Manini et al. (2007). The intensity was not reported for all studies; thus, it was not possible to show the mean intensity used in the studies. In turn, the exercises performed in FT sessions are very different between groups, with similarities between the two studies by Resende Neto et al. (2019a), Resende Neto et al. (2019b).

As for the evaluation methods used, the studies analyzed in this review mostly involved electrical bioimpedance systems as an evaluation instrument for verifying the adaptations provided in body composition, presenting results of percentage of fat mass, percentage of lean mass, total fat mass or total lean mass (Wiszomirska et al., 2015; Resende-Neto et al., 2019a; Resende-Neto et al., 2019b; Mile et al., 2021), except for the study by Manini et al. (2007) that used the DEXA method and presented values of appendicular fat mass, appendicular lean mass and lean to fat ratio.

Most studies included measurements of total body fat and total lean body mass. We can observe a mean decrease in fat mass of 4.25% and a mean increase of 1,60% in lean body mass. The weighted average [(effect size x sample population)/sample population] for the five studies demonstrated magnitude effect size changes of −0.20 to 0.12 for fat mass and lean mass respectively, characterized as small for fat mass and trivial for lean mass (Cohen, 1988). Only the study by Manini et al. (2007), Resende-Neto et al. (2019), and Mile et al. (2021) showed significant reductions in fat mass induced by FT. Only Wiszormiska et al. (2015) and Mile et al. (2021) studies showed significant increases in lean mass promoted by FT practice.

### 3.3 Methodological quality of included studies

When we performed the Assessment of Methodological Quality using the PEDro scale, only the studies of Resende Neto scored equal or higher than 6, qualifying as high-quality studies (Resende-Neto et al., 2019a; Resende-Neto et al., 2019b) (Table 2).

**TABLE 2:** PEDro Scale scores of the reviewed articles.

	Elegibility criteria specified	Random allocation	Concealed allocation	Groups similar at baseline	Subject blinding	Therapist blinding	Assessor blinding	Less than 15% dropouts	Intention-to-treat analysis	Between group statistical comparisons	Point measures and variability data	PEDro score total
Manini et al. (2007)	**-**	**+**	**-**	**+**	**-**	**-**	**-**	**-**	**-**	**+**	**+**	4
Wiszormiscka et al. (2015)	**-**	**-**	**-**	**-**	**-**	**-**	**-**	**-**	**-**	**+**	**+**	2
Resende- Neto et al., 2019a	**+**	**+**	**-**	**+**	**-**	**-**	**+**	**-**	**-**	**+**	**+**	6
Resende- Neto et al., 2019b	**+**	**+**	**-**	**+**	**-**	**-**	**+**	**+**	**-**	**+**	**+**	7
Mile et al. (2021)	**+**	**-**	**-**	**+**	**-**	**-**	**-**	**+**	**-**	**+**	**+**	5

## 4 Discussion

The present review found only a few studies in the scientific literature with high methodological quality that provide data on this theme. In this context, FT interventions with a duration between five and 6 months, seem to reduce the total fat mass. Meanwhile, studies with shorter interventions, lasting 10–12 weeks, have no consensus on FT effects on fat composition. Although some studies point to the reduction of fat mass promoted by FT, the magnitude of change is considered small in four of the five studies analyzed in this review (Cohen, 1988). Similarly, the three studies incorporating less than 12 weeks of FT had trivial increases in lean mass while one of the two studies with more than 20 weeks of training showed a small magnitude improvement in lean mass. Thus, it is necessary to be cautious in interpreting the effects of FT on the body composition of older people since the studies found had low methodological quality and diverse findings.

By stimulating different energy pathways, FT produces high activity-induced energy expenditure causing fat tissue mobilization and lipid oxidation (Westerterp, 2018). The differences among the studies may be related to the lack of standardization of the intensity and duration of sessions used since this is an important variable in lipid oxidation that occurs at maximal rates when the intensity is maintained between −60 and 65% VO2 max (Hargreaves and Spriet, 2020). Another possible explanation for the divergent results with shorter interventions relies on the absence of nutritional control in the studies included in the review, necessary to verify the possible increasing energy intake, given the importance of nutritional intervention in body composition adaptations and health promotion (Makanae and Fujita, 2015).

One of the two studies using long-term interventions of between five and 6 months, showed a small but significant increase in lean mass (Wiszomirska et al., 2015; Mile et al., 2021). However, studies with shorter durations, between 10 and 12 weeks, did not demonstrate improvements in lean mass (Manini et al., 2007; Resende-Neto et al., 2019a; Resende-Neto et al., 2019b). Possible explanations for the difference between the results of the studies would be the difference in the total volume and duration of the interventions, important variables in promoting muscle hypertrophy (Krieger, 2010; Schoenfeld et al., 2019; Bernárdez-Vázquez et al., 2022). When we analyze the resistance training (RT) that already has the largest body of evidence in the literature, the effects of training volume on lean mass adaptation kinetics are still unclear in older people (Thomas et al., 2021). The literature indicates that the minimal time for young adults to achieve substantial hypertrophy with resistance training is approximately eight weeks (Damas et al., 2018), on the other hand, older people have lower protein synthesis and reduced activation of muscle satellite cells following exercise (Burd et al., 2013; Nederveen et al., 2020). Thus, greater training doses may be necessary promote hypertrophic alterations. Furthermore, according to the meta-regression of Borde et al. (2015), RT training periods between 50 and 53 weeks would be effective for increasing lean mass in healthy older people, presenting longer duration times than those presented by the studies analysed in this review. Although the literature has not yet established exact parameters for the minimal dose to improve lean mass in older people, the current suggested volume is at least 10 sets per week per muscle group (Santana et al., 2021).

From the studies in this analysis, we noticed a lack of standardization in the FT exercise stimuli offered, making it difficult to interpret the effects of FT on body composition. For example, Mile et al. (2021) provided a training protocol with squat, pull, and push patterns performed with suspension tape, diverging from the protocol of Manini et al. (2007), which used exercises similar to activities of daily living of older people, such as vacuuming a carpet and climbing a stair step. We can notice that almost all the studies involved presented session duration between 45 and 50 min, except for the study of Manini et al. (2007), which presented sections between 30 and 45 min. As for the weekly frequency, the studies presented variations between two and three times per week. In turn, the intensity was not reported in all articles. Thus, we cannot notice trends for session duration, weekly frequency, and intensity concerning the outcome of changes in body composition. Similarly, Marín-Cascales et al. (2018), in a recent meta-analysis on the effects of multicomponent training on body composition variables in older people, pointed out the diverse characteristics of training protocols applied to this public as a significant limitation to allow generalizations about the findings.

Regarding the evaluation methods used, the majority of the studies analyzed utilized electrical bioimpedance systems to provide information about the body composition (Wiszomirska et al., 2015; Resende-Neto et al., 2019a; Resende-Neto et al., 2019b; Mile et al., 2021). Although DEXA is considered the gold standard in body composition assessment, electrical bioimpedance presents itself as an attractive option, being less expensive and easy to transport and handle. Notwithstanding, both types of equipment tend to overestimate the lean mass values, also, to our best knowledge, the level of reproducibility of bioimpedance for this population is not clear in the literature (Bauer and Morley, 2020). In any case, the usefulness of bioimpedance for older people has already been presented as a possibility for body composition assessment, even collaborating in the diagnosis of pathologies such as sarcopenia (Akamatsu et al., 2022).

When analyzing the results, we noticed the predominance of women in the samples, being 100% for four of the analyzed studies (Wiszomirska et al., 2015; Resende-Neto et al., 2019a; Resende-Neto et al., 2019b; Mile et al., 2021) and 90.6% for the study by Manini et al., however, despite presenting men in its sample, it is valid to point out that in the group that performed FT, there were only women. From this perspective, data suggest that men seek fewer primary healthcare programs than women (Levorato et al., 2014). In addition, older women are more affected by the loss of lean mass and increase in body fat possibly due to reduced levels of endogenous estrogen (Janssen et al., 2000; Jafarinasabian et al., 2017). These differences alter the skeletal muscle metabolism during exercise, affecting the exercise responsiveness of women concerning increasing lean mass and reducing fat mass, making the findings of our review limited to older women (Hargreaves and Spriet, 2020; O’Bryan et al., 2022).

Furthermore, understanding that the maintenance of the stimulus is important to the promotion of chronic effects provided by the exercise, the adherence to the exercise program can also influence the results (Beaudry, 2015; Johnston et al., 2019). However, the studies reported here do not present the percentage of adherence of the participants, which may be a confounding factor for their findings. Another possible confounding factor is age. When analyzing the results, we should consider that the mean reported in the study by Manini et al. was about 10 years older than the other included studies. Considering that every 10 years there is a loss of 5% to 10% in the lean mass of older women (Westcott, 2012), this may represent differences in the values of body composition between the studies included.

We must interpret the data from this review with caution because few studies were included. Also, few studies presented good methodological quality. It highlights the need for new studies with improved methodological quality to understand the adaptations provided by FT on body composition in older people. In this pathway, we point out the lack of literature on randomized clinical trials that apply FT by properly controlling the variables of intensity, weekly frequency, session duration, and exercises. To better understand the necessary adaptation time for each variable, we suggest that these studies address different total duration times. It is also necessary to separate the analyses between older men and women, with the perspective of understanding the differences between genders in this adaptation.

## 5 Conclusion

Longer FT interventions, between five and six months, appear to reduce fat percentage with conflicting results regarding changes in lean mass in older women. In addition, studies with short interventions, between 10 and 12 weeks, shows conflicting results. It is also valid to point out the lack of scientific literature about studies of high methodological quality that analyze the effects of FT on the body composition of older people. Therefore, this review attempts to provide initial directions and observations to this gap in the literature.

## Data Availability

The raw data supporting the conclusion of this article will be made available by the authors, without undue reservation.
