# Supplementary material for: Is functional training an efficient approach to improve body composition in older people? A systematic review
Source: Front Physiol. 2023 Jun 19;14:1156088. doi: 10.3389/fphys.2023.1156088 (PMC10315661; doi:10.3389/fphys.2023.1156088)
Supplement: Supplementary file 1 [file DataSheet1.pdf]

Supplementary Material

Is functional training an efficient approach to improve body composition in older people? A systematic review

Marcos Raphael Pereira Monteiro [1,2], Alan Pantoja Cardoso [3], Antônio Gomes de Resende-Neto [4], Alan Bruno Silva Vasconcelos [1], Enilton Aparecido Camargo [1], Luis Alberto Gobbo [5], José Luis Maté-Muñoz [6], Juan Ramón Heredia-Elvar [7], David George Behm [8], Marzo Edir Da Silva-Grigoletto [1,3,4]*

1 Department of Physiology, Federal University of Sergipe, São Cristóvão, Brazil.

2 Department of Physiotherapy, Federal University of Sergipe, Lagarto, Brazil.

3 Department of Physical Education, Federal University of Sergipe, São Cristóvão, Brazil.

4 Department of Medicine, Federal University of Sergipe, São Cristóvão, Brazil.

5 Department of Physical Education, São Paulo State University, Presidente Prudente, Brazil.

6 Department of Radiology, Rehabilitation and Physiotherapy, Complutense University of Madrid.

7 Department of Physical Activity and Sports Science, Alfonso X El Sabio University, Madrid, Spain.

8 School of Human Kinetics and Recreation, Memorial University of Newfoundland, St. John's, Newfoundland and Labrador, Canada

*** Correspondence:**
Marzo Edir Da Silva-Grigoletto
medg@ufs.br

1 Search strategies utilized

Pubmed MEDLINE:

(("Functional Training") OR ("Functional Exercise") OR ("Functional Task Training") OR ("Functional Task Exercise")) AND ((Elderly) OR (Aged) OR (“Older Adults”) OR (“Older People”)) (randomized controlled trial[Publication Type] OR (randomized[Title/Abstract] AND controlled[Title/Abstract] AND trial[Title/Abstract]))

Scopus:

(TITLE-ABS-KEY (("Functional Training") OR ("Functional Exercise") OR ("Functional Task Training") OR ("Functional Task Exercise")) AND TITLE-ABS-KEY (("Elderly") OR ("Aged") OR ("Older Adults") OR ("Older People")))

Web of Science:

(ALL=(("Functional Training") OR (“Functional Exercise") OR ("Functional Task Training") OR ("Functional Task Exercise")) AND ALL=((Elderly) OR (Aged) OR ("Older Adults") OR (“Older People”)))

Cochrane Library:

#1 (“Functional Training” OR "Functional Exercise" OR "Functional Task Training" OR "Functional Task Exercise")

#2 ("Elderly" OR "Aged" OR "Older Adults" OR “Older People”)

#1 AND #2

Google Scholar:

(("Functional Training") OR ("Functional Exercise") OR ("Functional Task Training") OR ("Functional Task Exercise")) AND ((Elderly) OR (Aged) OR (“Older Adults”) OR (“Older People”))
